# Effects of lead exposure on blood electrical impedance spectroscopy of mice

**DOI:** 10.1186/s12938-021-00933-0

**Published:** 2021-10-07

**Authors:** Binying Yang, Jia Xu, Shao Hu, Boning You, Qing Ma

**Affiliations:** 1 Ninghai First Hospital, Ninghai, 315600 Zhejiang China; 2 School of Medicine, Ningbo University, Ningbo, 315211 Zhejiang China

**Keywords:** Lead-exposed, Blood, Electrical impedance spectroscopy, Characteristic frequency, Constant phase element

## Abstract

**Background:**

Lead is a nonessential heavy metal, which can inhibit heme synthesis and has significant cytotoxic effects. Nevertheless, its effect on the electrical properties of red blood cells (RBCs) remains unclear. Consequently, this study aimed to investigate the electrical properties and the electrophysiological mechanism of lead exposure in mouse blood using Electrical Impedance Spectroscopy (EIS) in 0.01–100 MHz frequency range. Data characteristic of the impedance spectrum, Bodes plot, Nyquist plot and Nichols plot, and Constant Phase Element (CPE) equivalent circuit model were used to explicitly analyze the differences in amplitude–frequency, phase–frequency, and the frequency characteristics of blood in electrical impedance properties.

**Results:**

Compared with the healthy blood in control mice, the changes in blood exposed to lead were as follows: (i) the hematocrit decreased; (ii) the amplitude–frequency and phase–frequency characteristics of electrical impedance decreased; (iii) the characteristic frequencies (*f*_0_) were significantly increased; (iv) the electrical impedance of plasma, erythrocyte membrane, and hemoglobin decreased, while the conductivity increased. (v) The pseudo-capacitance of cell membrane (CPE_Tm) and the intracellular pseudo-capacitance (CPE-Ti) were decreased.

**Conclusions:**

Therefore, EIS can be used as an effective method to monitor blood and RBC abnormalities caused by lead exposure. The electrical properties of the cells can be applied as an important observation in the evaluation of the toxic effects of heavy metals.

## Background

Lead is a heavy metal material that has been widely applied in pottery, lead welding, dyes, and cosmetics [1]. Exposure to the high concentration of lead may cause damage to the nervous system, digestive system, blood system, kidney, and other organs [2–4]. A new analysis by the Institute for Health Metrics and Evaluation (IHME) has estimated that 815 million children worldwide have a high concentration of lead in their bloodstream [5].

Hematological studies have shown that 95% of blood lead is bound to erythrocyte membrane and hemoglobin, while the rest (5%) is found in the plasma. Lead can affect heme synthesis by interfering with porphyrin metabolism disorder. This can eventually cause the compensatory proliferation of erythroblasts in bone marrow, increase reticulocyte and alkaline granulocyte and decrease of the numbers of erythrocytes and leukocytes in the blood, which in turn lead to hemolytic anemia [6]. Ahyayauch et al*.* measured the membrane properties of rat erythrocytes and erythrocyte membrane ghosts by either chronic or acute Pb^2+^ treatments and found that 1.8 μM Pb^2+^ could directly increase cell membrane permeability, thereby increasing hemolysis [7]. However, despite these documented molecular insights into the lead on blood or immunocytes, there is still no report on the electrical properties of lead exposure in mouse blood. The electrophysiological mechanism of lead-exposed RBCs remains unclear and needs to be further explored.

Electrical impedance spectroscopy of cells is widely accepted as a label-free, non-invasive, and quantitative analytical method for the assessment of live biological cells’ electrical properties and heterogeneity [8]. Kim et al. designed a microfluidic-based physiometer capable of measuring the electrical characteristics of the blood, i.e., the cytoplasm resistance (*R*_cytoplsm_), plasma resistance (*R*_plasma_), and RBC membrane capacitance based on EIS. Combined with image processing, the effects of the hematocrit and RBC deformability on the whole blood viscosity have been previously reported [9]. In their systematical analysis, Babić et al*.* pointed out that all-natural polyphenol flavonoids share a common non-specific mechanism of platelet activation and aggregation inhibition by the impedance spectrum and flow cytometry, which is related to their lipophilicity and membrane stability. It is of great significance to guide the formation and prevention of thrombosis in cardiovascular diseases [10]. With the development of impedance spectroscopy technology, EIS has been widely used in hematology detection, clinical diagnosis, disease mechanism exploration, and drug research and development.

In this study, we investigated the electrical properties and the electrophysiological mechanism of lead exposure in mouse blood using a lead-exposed mouse model and AC impedance measurement technology. Impedance spectrum bodes diagram, Nyquist diagram, Nichols diagram, and CPE-equivalent circuit model were comprehensively analyzed to establish the evaluation of blood electrophysiological parameters on lead exposure. This study provides a novel research method for the application of impedance spectroscopy in clinical diagnosis and lead poisoning treatment.

## Results

### Effect of lead exposure on electrical impedance spectroscopy and Nyquist plots of blood

**Fig. 1:**
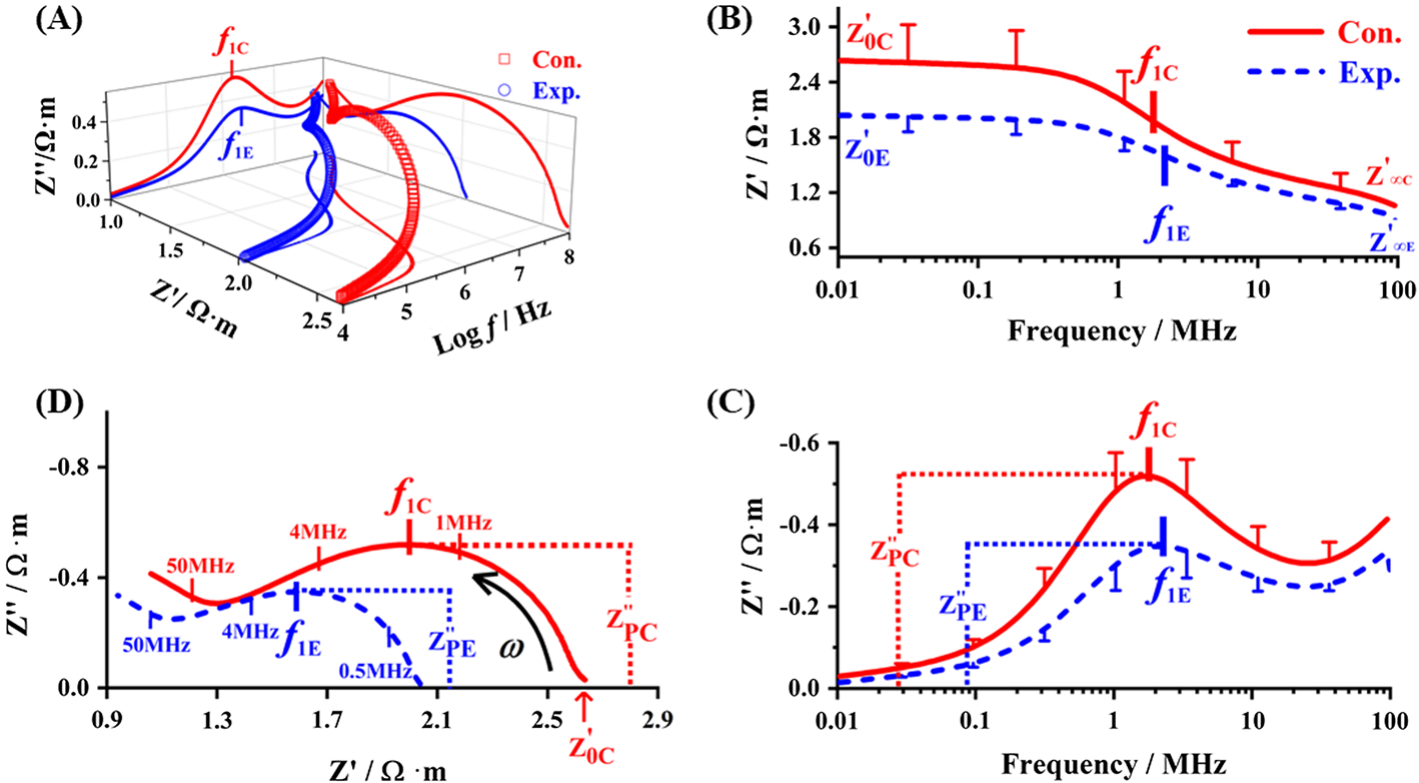
Effects of lead exposure on the real and imaginary parts of electrical impedance spectrum and Nyquist plot of blood in mice. **A** The 3D stereograph of blood impedance spectrum; **B** the $Z^{\prime}\left( f \right)$ curve; **C** the $Z^{\prime\prime}\left( f \right)$ curve; **D** Nyquist plots. In the 3D curve and axial plane projection of **A**, hollow squares and solid lines represent the measured spectrum, *f* − $Z^{\prime}$ projection (*x*–*y* axis, **B**), *f* − *θ* projection (*y*–*z* axis, **C**) and $Z^{\prime} - Z^{\prime\prime}$ projection (*x*–*z* axis, **D**) of control group (Con., red) and lead-exposed group (Exp., blue). In **B**–**D**, the measured spectra of control and exposure group are represented by solid curves (red) and dotted curves (blue), respectively

The blood Hct of the exposure group (37.52 ± 3.67%) was 9.66% (*p* < 0.05) lower than that of the control group (41.53 ± 3.6%), which suggested that the number of RBCs decreased due to lead exposure. Figure 1A clearly shows the relationship between the 3D spectrum and 2D projection (Fig. 1B–D) of electrical impedance spectrum in lead-exposed blood. The real part of the Impedance–Frequency spectrum [$Z^{\prime}\left( f \right)$ curve] is presented in Fig. 1B. The limit of the real part of impedance at low frequency $\left( {Z^{\prime}_{{0{\text{C}}}} \;{\text{and}}\;Z^{\prime}_{{0{\text{E}}}} } \right)$ reflects the electrical properties of the blood plasma, and RBCs’ suspension shows high impedance characteristic of capacitance at low frequencies. With increasing excitation frequency from 0.1 to 10 MHz, the capacitance reactance of the cell membrane and the impedance of RBCs’ suspension begin to decrease due to incomplete polarized RBCs. The characteristic frequency *f*_0_ performs as $\sqrt {f_{1} \times f_{2} }$ [11]. At high electric field frequencies (> 10 MHz), there is insufficient time for the cells to become polarized, and consequently, the current flows into the intracellular fluid. The $Z^{\prime}\left( f \right)$ curve continues as $Z^{\prime}$ decreases to $Z^{\prime}_{{\infty {\text{C}}}}$, which performs the capacitive short circuit of the membrane. Thus, the limit of the real part of impedance ($Z^{\prime}_{{\infty {\text{C}}}}$) represents the electrical characteristics of intracellular hemoglobin [12]. After lead exposure, the $Z^{\prime}\left( f \right)$ spectrum of exposure group shifted down to the low impedance region, which leads to the decreasing of $Z^{\prime}_{{0{\text{E}}}}$ and $Z^{\prime}_{{\infty {\text{E}}}}$ for characterization of plasma and hemoglobin impedance. The resistance of blood decreased is consistent with the EIS performance of glucose-6-phosphate dehydrogenase deficiency [13]. Figure 1C shows the frequency spectrum $Z^{\prime\prime}\left( f \right)$ of the imaginary part of the blood impedance. Below the 0.1 MHz, the $Z^{\prime\prime}$ value for the blood is very small and stable, with a value close to zero. From 0.1 to 10 MHz, a single characteristic peak is formed at the interface of the cell membrane, and plasma follows from the polarization loss of the induced charges. There are two parameters to the characteristic peak: the peak of the imaginary part of impedance $\left( {Z^{\prime\prime}_{{{\text{PC}}}} } \right)$ and 1st characteristic frequency ($f_{{1{\text{C}}}}$). The curve showed a concave-like increasing tendency from 10 to 100 MHz, with the trend of an upturned tail rise at the higher band, which also appeared in the EIS of frog-blood [14]. While the hump-shaped curve exhibits a downward shift with the value of $Z^{\prime\prime}_{{{\text{PE}}}}$ decreases and $f_{{1{\text{E}}}}$ increases of exposure group. The electrical impedance spectroscopy Nyquist plots present a semicircle arc at low frequency and an individual semicircle arc with an upturned tail rise at the higher band stretched from right to left (Fig. 1D). The center of the semicircle below the abscissas, together with a graphical definition of the vertices and the height of the semicircle present $f_{{1{\text{C}}}}$ and $Z^{\prime\prime}_{{{\text{PC}}}}$, respectively. Compared with the control group, the limit of the real part of impedance at low frequency ($Z^{\prime}_{{0{\text{E}}}}$), peak of the imaginary part of impedance $\left( {Z^{\prime\prime}_{{{\text{PE}}}} } \right)$, the radius and area of arc were decreased, thus revealing that lead exposure induced decreased resistance in the blood of mice.

### Effect of lead exposure on Bode plots and Nichols plots of blood

**Fig. 2:**
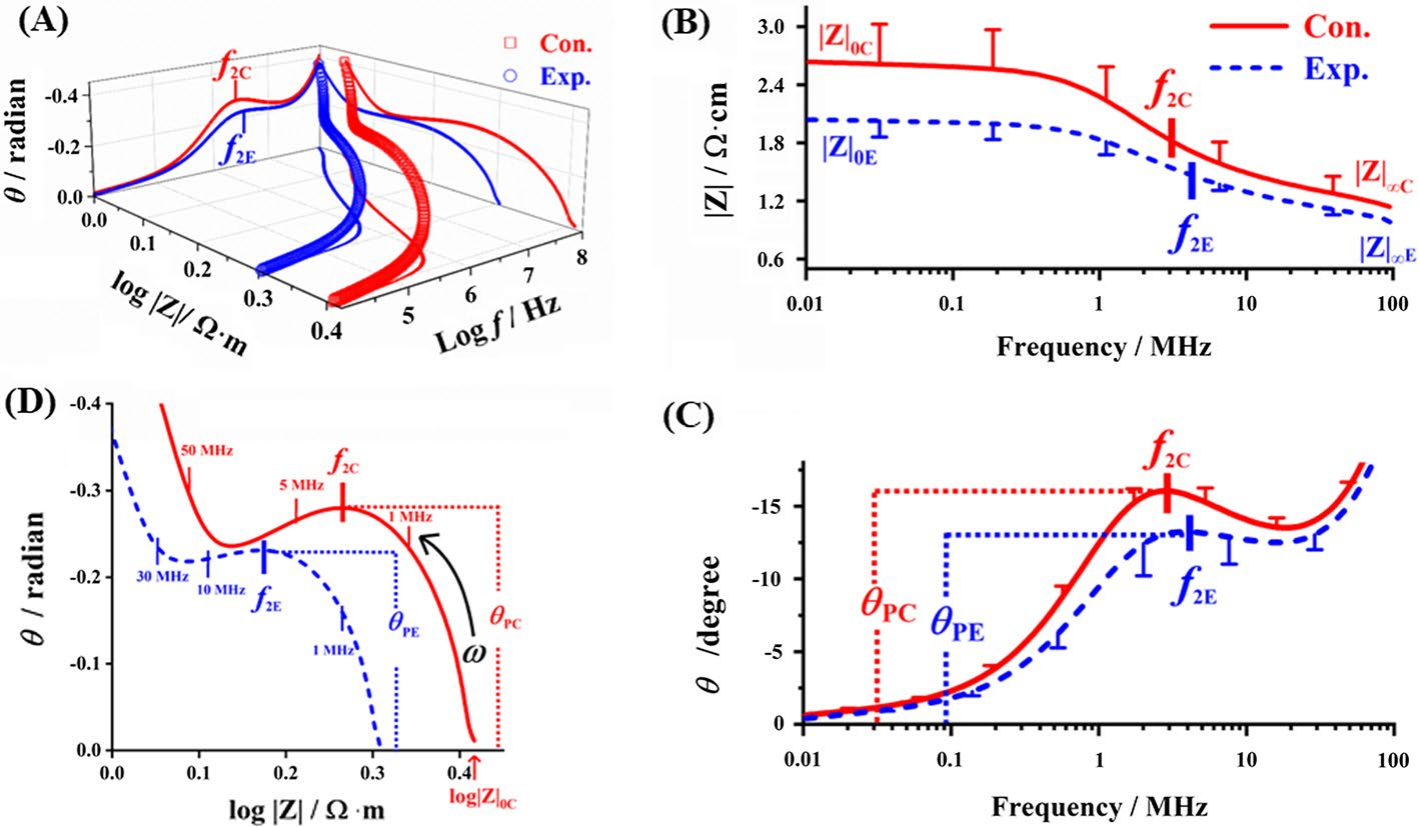
Effect of lead exposure on the Bode and Nichols plots of mice blood. **A** Amplitude–phase–frequency 3D stereogram, **B** amplitude–frequency curves; **C** phase–frequency curves; **D** Nichols plots. Labels same as in Fig. 1

The current flowed through the plasma, erythrocyte membrane, and hemoglobin region as the external electric field increases. The amplitude–phase–frequency 3D stereogram (Fig. 2A) represents the impedance changes before and after lead exposure to blood. Compared with the control group, the amplitude–frequency curves of Bode plots showed a downshift overall trend (Fig. 2B). Impedance amplitude at low frequency (|*Z*|_0E_ = 2.03 ± 0.17 Ω m) and the impedance amplitude increment (*∆*|*Z*|_E_ = 1.08 ± 0.16 Ω m) of exposure group had a significant decrease of 21.32% and 29.87% compared to control (|*Z*|_0C_ = 2.58 ± 0.33 Ω m, *∆*|*Z*|_C_ = 1.54 ± 0.22 Ω m), respectively. Moreover, the impedance amplitude at high frequency (|*Z*|_∞, E_ = 1.00 ± 0.05 Ω m) was reduced by 10.71% but was not statistically significant. The results indicated that blood exposure to lead induced variable degrees reduction of the electrical impedance in plasma, erythrocyte membrane, and hemoglobin. Moreover, the electrical impedance of extracellular plasma and cell membrane was sensitive to lead exposure. Likewise, the phase–frequency curves of Bode plots showed a significant downward shift compared with the control group (Fig. 2C). The peak of phase angle (deg) of exposure group ($\theta_{{{\text{PE}}}}$ = − 13.23 ± 1.96) was reduced by 17.00%, and the 2nd characteristic frequency ($f_{{2{\text{E}}}}$ = 4.96 ± 2.47 MHz) increased by 76.51% compared with the control group ($\theta_{{{\text{PC}}}}$ = − 15.94 ± 0.85, $f_{{2{\text{C}}}}$ = 2.81 ± 0.23 MHz) significantly. The Nichols plots present a semicircle with an upturned tail rise curve from the low- to the high-frequency band, which translated to the left with the increasing of the applied AC electrical field (Fig. 2D). This is accompanied by the reduction of logarithm of low-frequency impedance amplitude (log|*Z*|_0E_), peak of phase angle ($\theta_{{{\text{PE}}}}$/rad), the radius and area of arc, and the increasing of 2nd characteristic frequency ($f_{2}$) under the lead exposure blood. Based on the above data, the EIS parameters of blood ($Z^{\prime\prime}_{{\text{P}}}$, $\theta_{{\text{P}}}$ and $f_{0}$) are reliable and valid factors for assessing the electrical properties of erythrocyte membrane, which could be used to identify and characterize the charge/discharge processes.

### Effect of lead exposure on impedance parameters of blood

**Fig. 3:**
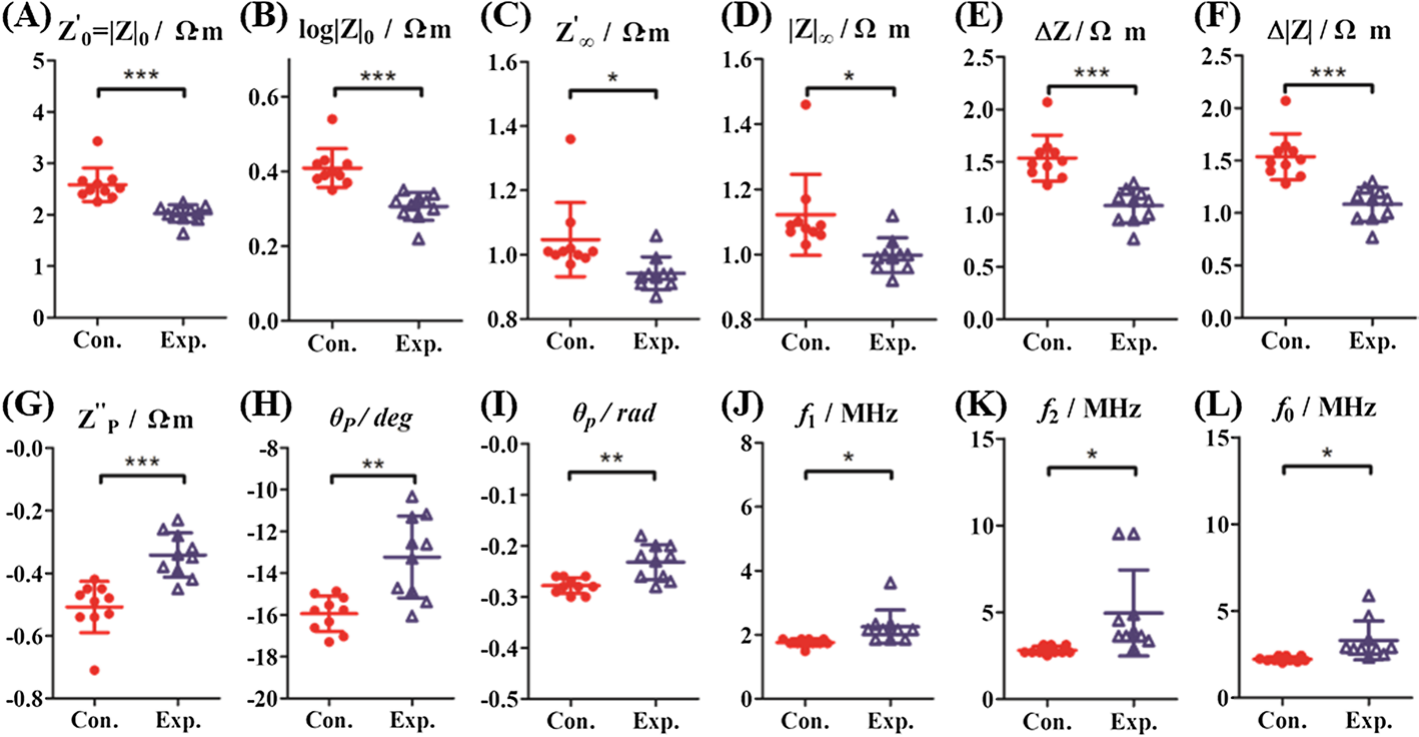
Effect of lead exposed on electrical impedance spectral parameters of blood. **A** Impedance amplitude at low frequency (|*Z*|_0_); **B** logarithm of low-frequency impedance amplitude (log|*Z*|_0_); **C** the limit of the real part of impedance at high frequency ($Z^{\prime}_{\infty }$); **D** impedance amplitude at high frequency (|*Z*|_∞_); **E** real-part increment of electrical impedance (${\Delta }Z = Z^{\prime}_{0} - Z^{\prime}_{\infty }$); **F** impedance amplitude increment (Δ|*Z*| = |*Z*|_0_ − |*Z*|_∞_); **G** peak of imaginary part of impedance ($Z^{\prime\prime}_{{\text{p}}}$); **H**, **I** peak of phase angle (*θ*_p_/deg and *θ*_p_/rad); **J** the 1st characteristic frequency (*f*_1_); **K** the 2nd characteristic frequency (*f*_2_); **L** characteristic frequency ($f_{0} = \sqrt {f_{1} \times f_{2} }$). Solid circles represent control group (Con.) and hollow triangles represent lead-exposed group (Exp.). *Significance at *p* < 0.05, **significance at *p* < 0.01 and ***significance at *p* < 0.001, ns indicates no significant difference

According to the results from electric impedance parameters analysis (Fig. 3 and Table 1), there were three changes of lead exposure on blood: (i) the exposure group possessed a reduction of the real part and magnitude of the impedance parameters ($Z^{\prime}_{0}$, $Z^{\prime}_{\infty }$, Δ*Z*, |*Z*|_0_, |*Z*|_∞_, Δ|*Z*|, log|*Z*|_0_) by 21.80%, 10.48%, 29.87%, 21.32%, 10.71%, 29.87%, and 33.33%, respectively. (ii) Results expressed a significant decrease of the imaginary part and phase angle parameters ($Z^{\prime\prime}_{{\text{P}}}$, *θ*_p_/deg and *θ*_p_/rad) by 26.83%, 17.00%, and 17.86%, respectively. (iii) Results showed a significant increase in frequency parameters ($f_{1}$, $f_{2}$ and $f_{0}$) by 27.84%, 76.51% and 48.65%, respectively. These changes of electrical properties are the intuitive feature of the cell membrane and intracellular biochemical reactions by lead exposure.

**Table 1:** The effect of lead exposure on the properties of electrical impedance spectra of mice blood

Parameters	Symbol/unit	Control (*n* = 10)	Experimental (*n* = 10)	Rate of change (%)
Low-frequency limit of real part of impedance	$Z^{\prime}_{0}$/Ω m	2.58 ± 0.33	2.02 ± 0.17***	− 21.80
High-frequency limit of real part of impedance	$Z^{\prime}_{\infty }$/Ω m	1.05 ± 0.12	0.94 ± 0.05*	− 10.48
Real-part increment of electrical impedance	∆*Z*/Ω m	1.54 ± 0.22	1.08 ± 0.16***	− 29.87
Impedance amplitude at low frequency	|*Z*|_0_/Ω m	2.58 ± 0.33	2.03 ± 0.17***	− 21.32
Impedance amplitude at high frequency	|*Z*|_∞_/Ω m	1.12 ± 0.12	1.00 ± 0.05*	− 10.71
Impedance amplitude increment	∆|*Z*|/Ω m	1.54 ± 0.22	1.08 ± 0.16***	− 29.87
Logarithm of low-frequency impedance amplitude	log|*Z*|_0_/Ω m	0.41 ± 0.05	0.30 ± 0.04***	− 33.33
Peak of imaginary part of impedance	$Z^{\prime\prime}_{{\text{p}}}$/Ω m	− 0.51 ± 0.08	− 0.34 ± 0.07***	− 26.83
Peak of phase angle (deg)	*θ*_p_/deg	− 15.94 ± 0.85	− 13.23 ± 1.96**	− 17.00
Peak of phase angle (rad)	*θ*_p_/rad	− 0.28 ± 0.01	− 0.23 ± 0.03**	− 17.86
The 1st characteristic frequency	*f*_1_/MHz	1.76 ± 0.12	2.25 ± 0.52*	27.84
The 2nd characteristic frequency	*f*_2_/MHz	2.81 ± 0.23	4.96 ± 2.47*	76.51
Characteristic frequency	*f*_0_/MHz	2.22 ± 0.15	3.30 ± 1.12*	48.65

^*^p < 0.05, **p < 0.01, ***p < 0.001, compared with the control group

### Effect of lead exposure on the parameters of a CPE-equivalent electrical circuit in blood

**Fig. 4:**
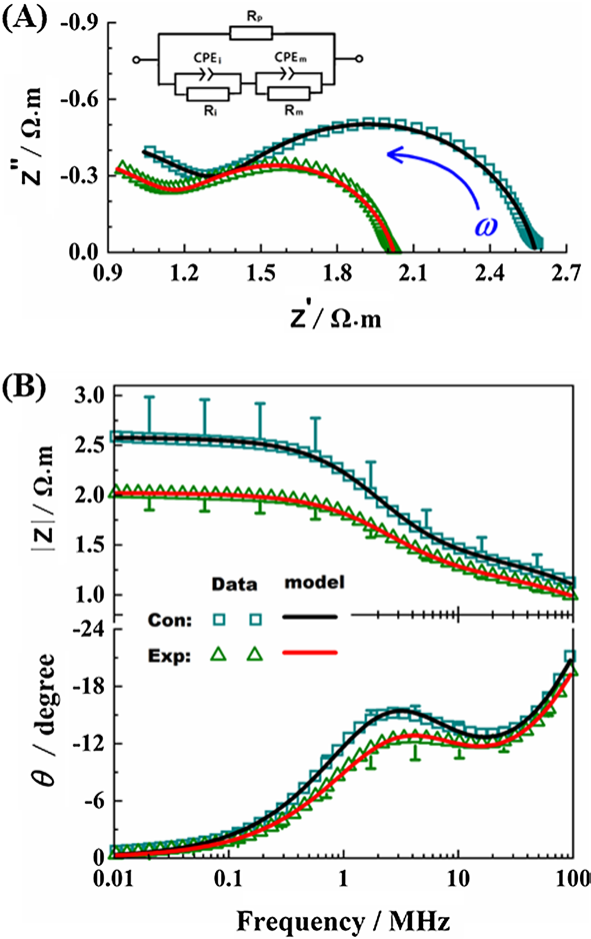
Effect of lead exposure on equivalent circuit parameters of mouse blood. **A** CPE-equivalent electrical circuit model of blood and Nyquist plot, *R*_p_ represents plasma resistance, CPEi intracellular constant phase element, *R*_i_ cell interior resistance, CPEm constant phase element of cell membrane and *R*_m_ cell membrane resistance. **B** Bode plot

The cell structural parameters (Table 2) were established based on the CPE-equivalent electrical circuit model (Fig. 4), which was obtained through the curves fitting of blood electrical impedance spectrum observation data by Zview2 software. Compared with control, *R*_p_, *R*_m_, CPE_Tm, and CPE-Ti were decreased by 18.76% (*p* < 0.001), 42.13%, 20.45%, and 29.05%, respectively.

**Table 2:** The values of electric components of the established equivalent circuit model

Parameters	Symbol/unit	Control (*n* = 10)	Experimental (*n* = 10)	Rate of change (%)
Hematocrit	Hct/%	41.53 ± 3.6	37.52 ± 3.67*	− 9.66
Plasma resistance	*R*_p_/Ω m	2.48 ± 0.14	2.02 ± 0.16***	− 18.76
Pseudo-capacitance of cell membrane	CPE___Tm/${\text{nF}}\;{\text{s}}^{{\upalpha _{{\text{m}}} - 1}}$	290.77 ± 98.26	231.3 ± 132.24	− 20.45
Dispersion coefficient of cell membrane	${\upalpha }_{{\text{m}}}$	0.83 ± 0.02	0.84 ± 0.04	1.66
Cell membrane resistance	*R*_m_/MΩ m	3.60 ± 2.63	2.08 ± 0.95	− 42.13
Intracellular pseudo-capacitance	CPE___Ti/${\text{nF}}\;{\text{s}}^{{\alpha_{{\text{i}}} - 1}} { }$	89.55 ± 23.85	63.53 ± 54.87	− 29.05
Intracellular dispersion coefficient	${\upalpha }_{{\text{i}}}$	0.751 ± 0.011	0.79 ± 0.04	4.6
Intracellular resistance	*R*_i_/Ω m	2.58 ± 0.26	2.56 ± 0.41	− 0.79
Chi-squared	*χ*^2^	(1.89 ± 0.55)10^–4^	(2.44 ± 1.66)10^–4^	
Sum of Sqr	∑*χ*^2^	(4.58 ± 1.3310^–2^	(5.92 ± 4.0410^–2^	

*p < 0.05, ***p < 0.001, compared with the control group

## Discussion

Based on our results, the lower Hct of lead exposure in mouse blood was consistent with lead-exposed blood of different species, such as *Algerian Mice* [15] *Apodemus sylvaticus* [16], *Parus major* [17], adults [18], and children [19]*.* As shown in the real and imaginary part of impedance magnitude (Figs. 1B and 2B) of blood, many ions have time to reach the cell membrane before the electric field is reversed at the low frequency. On the contrary, few ions have time to polarize the cell membranes before the field is reversed under the high frequency, this results in a negligible contribution towards the capacitance [20, 21]. The polarized RBC membrane has capacitor characteristics; the capacitance reactance [1/(*ωC*_m_)] decreases with increasing frequency. As current is hindered by the high impedance of RBCs membrane, it consequently flows through the extracellular plasma, which is expected due to the low impedance properties. Overall, the decreasing trend of the real part of impedance ($Z^{\prime}$) or amplitude generally occurred between 0.1 and 10 MHz, which is typically referred to as *β* dispersion. There are two sub-relaxations in *β* dispersion; the 1st and 2nd characteristic frequencies (*f*_1_ and *f*_2_ in Figs. 1C and 2C, respectively), which come from the existence of plasma–cytomembrane and cytomembrane–hemoglobin interfacial polarizations, respectively. Accompanied by an external electric field, the accumulation of interface charge and the formation of interface polarization phenomena occur due to the interface hindering the charge transfer [22]. Similarly, blood exposure to lead induced variable degrees reduction of the electrical impedance in plasma, erythrocyte membrane, and hemoglobin, as shown in Bode plots and Nichols plots.

Notably, we introduce here an estimation protocol based on a multiparameter method of RBCs. $Z^{\prime}_{0}$ and |*Z*|_0_ reflect extracellular impedance properties, Δ*Z*, Δ|*Z*| and $f_{0}$ reflect cell membrane impedance properties, $Z^{\prime}_{\infty }$ and |*Z*|_∞_ reflect intracellular impedance properties. Under the long-term lead exposure, the Na^+^–K^+^ ATPase activity of erythrocyte membrane was inhibited, which induced an imbalance of Na^+^–K^+^ ion homeostasis in RBCs [23]; the formations of insoluble lead phosphate were synthesized from lead chelating phosphate of the erythrocyte membrane, which may lead to cell hemolysis by increasing the RBC membrane fragility and permeability [7, 24]. Consistent with G-6-PD deficiency of RBCs, the changes of biochemical and electrophysiological characteristics were the main factors that induced the significant decrease in RBC numbers and mass, declined hematocrit (Hct), increased conductivity, and reduced cell impedance [25]. Similarly, we speculate that the changes of phase characteristics and frequency parameters of RBCs induced by lead exposure were due to the weakening of the barrier effect of the high permeability cell membrane. Lead is more likely to interact with δ-aminolevulinic acid dehydratase (ALA-D), xanthinogen oxidase (XOD) and iron chelatase (FC) by inhibiting the synthesis of heme and cytochrome [26–28] thus leading to the decrease of hemoglobin, the phosphatidylserine exposure of membrane surface, the shrinkage of erythrocyte [29] and decrease of volume, as well as the appearance of anemia-like morphological changes [27, 28], eventually causing a significant increase of the characteristic frequency (*f*_1_, *f*_2,_ and *f*_0_) of RBCs [30].

More importantly, given that these feature parameters defined reference value for blood electrophysiology, future research efforts should explicitly analyze the differences in amplitude–frequency, phase–frequency, and the frequency characteristics of blood in electrical impedance properties. Encouragingly, Constant Phase Element (CPE) equivalent circuit model displayed inspiring curve fitting effect on observation data. The results were consistent with glucose-6-phosphate dehydrogenase (G-6-PD)-deficiency anemia [13]. For sickle anemia, the increased number of denatured hemoglobin that binds to the intracellular side of the cell membrane in RBCs of anemia [31, 32], which increases the membrane-associated heme and free iron leading to a lower *C*_m_ [33]. Our results further support the above previous study. More importantly, our methods provide an effective method (EIS), and a set of critical and important parameters for toxicological evaluation of heavy metals in blood.

## Conclusions

In summary, this study used the electrical impedance spectrum, Bode plots, Nyquist plots, and Nichols plots data analysis to confirm that lead exposure can reduce the Hct, decrease the impedance and phase angle characteristics, and increase the first and second characteristic frequencies of blood in mice. These results provided data support and new diagnosis and treatment methods for the hematotoxicity and the potential electrophysiological mechanism of lead exposure. Further, the present study focuses on the electrical properties of RBC suspension using Electrical Impedance Spectroscopy, while the electrical characteristics and microstructural alterations of single cells exposed in Pb have not been deeply studied and explained. Single-cell impedance analysis based on model analysis and parameter extraction deserves in-depth study, which will have more profound research value and reference significance.

## Methods

### Subjects and blood collection

A total of 20 ICR mice, weighing 27.5 ± 5.1 g on average, were provided by the experimental animal center of Ningbo University. All the animals were housed in an environment with a temperature of 22 ± 1 °C, relative humidity of 50 ± 1%, and a light/dark cycle of 12/12 h. All animal studies (including the mice euthanasia procedure) were done in compliance with the regulations and guidelines of Ningbo University institutional animal care and conducted according to the AAALAC and the IACUC guidelines.

The exposure group (*n* = 10) was gavaged with 0.5 ml PbA (250 mg/kg bw) per 24 h for 4 weeks; at the same time, the control group (*n* = 10) was treated with normal saline through identical administration. Blood samples were collected by orbital sinus puncture into heparinized microhematocrit tubes from all mice by diethyl ether anesthesia; blood impedance and hematocrit values were directly determined after pretreatment or centrifugation. Lead acetate (PbA) of AR grade and all other reagents were purchased from Merck (Life Sciences Co., Ltd) unless otherwise stated.

### Hematocrit and impedance measurement

Hematocrit (Hct) was measured with microhematocrit capillary tubes (1.5 mm outer diameter, 75 mm length; surgical instruments factory of Shanghai Medical Instruments Co., Ltd) in a bench-top hematocrit centrifuge (Haematokrit 210, Germany) for 5 min at 11,000 rpm. The hematocrit was calculated using the following equation [34]: Hematocrit (%) = red blood cells volume/blood total volume.

The amplitude |*Z*| and the phase angle *θ* of blood were measured by Agilent 4294A Impedance Analyzer (USA), equipped with Agilent 42942A terminal adapter and an Agilent 16192A parallel electrode SMD test fixture; analyzers were controlled by a Lenovo computer. Measurements were made at room temperature. Dispersion characteristic was measured in the frequency range 0.01–100 MHz with 124 frequency points. Each run was taken three times, and the data points were the average of these runs. The sample cell was made of clear Plexiglas tube and consisted of a chamber with two parallel platinum plates embedded on both sides, which was customized according to size as follows [14] 7.7 mm electrode diameter, 8.24 mm distance between electrode pairs, 46.57 mm^2^ well area, and 0.38 ml sample volume. The complex impedance was expressed as $Z = \left| Z \right| \times e^{ - j\theta } = Z^{\prime} + jZ^{\prime\prime}$. The real ($Z^{\prime}$) and imaginary ($Z^{\prime\prime}$) impedance was calculated as $Z^{\prime} = \left| Z \right| \times \cos \theta$ and $Z^{\prime\prime} = \left| Z \right| \times \sin \theta$, respectively.

### CPE-equivalent circuit analysis

Importantly, the RBCs may not be an “ideal” capacitor, as commonly assumed in the standard electrical elements. The constant phase element (CPE) model provided the best fit in terms of accuracy for the experimental data [35]. Using this model (Fig. 4A), the cell membrane and intracellular itself have their own resistance (respectively, *R*_m_, *R*_i_) and pseudo-capacitance (CPE_Tm, CPE-Ti), while the plasma is purely resistive (*R*_p_). The impedance in terms of the parallel/series combinations of elements of the CPE-equivalent circuit is given by

1 $$Z = Z_{{\text{p}}} \parallel \left[ {\left( {Z_{{{\text{CPEm}}}} \parallel Z_{{\text{m}}} } \right) + \left( {Z_{{{\text{CPEi}}}} \parallel Z_{{\text{i}}} } \right)} \right],$$

where *Z*_*k*_ = *R*_*k*_, for *k* ∈ (p, m, i), $Z_{k} \left( s \right) = \left( {s^{{\alpha_{l} }} \cdot{\text{CPE}}\_{\text{T}}_{l} } \right)^{ - 1}$ for* l* ∈ (m, i) while 0 < CPE_T < 1. In addition, *α* = CPE_P_. Therefore, the impedance of the equivalent circuit model using fractional circuit theory [36] becomes

2 $$Z\left( s \right) = \left\{ {\frac{1}{{R_{{\text{p}}} }} + \left[ {\frac{1}{{\left( {s^{{\alpha_{{\text{m}}} }} } \right) \cdot {\text{CPE}\_{\text{Tm}}} + \frac{1}{{R_{{\text{m}}} }}}} + \frac{1}{{\left( {s^{{\alpha_{{\text{i}}} }} } \right) \cdot {\text{CPE-}{\text{Ti}}} + \frac{1}{{R_{{\text{i}}} }}}}} \right]^{ - 1} } \right\}^{ - 1} ,$$

where *s* = *jω*. The complex impedance of (2) can be calculated using the replacement $s^{{\alpha_{l} }} = \omega^{{\alpha_{l} }} \left[ {{\text{cos}} \left( {\frac{{\pi \cdot \alpha_{l} }}{2}} \right) + j\sin \left( {\frac{{\pi \cdot \alpha_{l} }}{2}} \right)} \right]$. Parameters in Table 2 were obtained by automatic curve fitting of blood impedance spectrum data with Zview2 Software (Reference website: https://softadvice.informer.com/Z_View_Software_Free_Download.html).

### Statistical analysis

All values were expressed as the means ± standard deviation (SD). The significance of the differences between each value presented by the Control and Lead exposure group was evaluated by the Student *t* test using SPSS 12.0 software. A *p* value < 0.05 was considered to be statistically significant.

## Data Availability

The datasets generated in the current study are not publicly available due to the ethical restrictions preventing public sharing of data. A non-identified set may be requested after approval from the Review Board of the Institution. Requests for the data may be sent to the corresponding author.

## Abbreviations

RBCs: Red blood cells
EIS: Electrical impedance spectroscopy
CPE: Constant phase element
IHME: Institute for Health Metrics and Evaluation
*R*_cytoplsm_: Cytoplasm resistance
*R*_plasma_, * R*_p_: Plasma resistance
Hct: Hematocrit
ALA-D: δ-Aminolevulinic acid dehydratase
XOD: Xanthinogen oxidase
FC: Iron chelatase
PbA: Lead acetate
